# Do admission glucose levels independently predict coagulopathy in multiple trauma patients? A retrospective cohort analysis

**DOI:** 10.1007/s00068-023-02405-7

**Published:** 2024-02-14

**Authors:** Jorge Mayor, Pascal Gräff, Vera Birgel, Jan-Dierk Clausen, Tarek Omar-Pacha, Gökmen Aktas, Stephan Sehmisch, Philipp Mommsen

**Affiliations:** 1 Department of Trauma Surgery, Hannover Medical School, Carl-Neuberg-Straße 1, 30625 Hannover, Germany; 2 Hannover Medical School, Institute for Epidemiology, Social Medicine and Health Systems Research, Carl- Neuberg-Straße 1, 30625 Hannover, Germany

**Keywords:** Coagulopathy, Multiple trauma, Admission blood glucose, Hyperglycaemia

## Abstract

**Background:**

Coagulopathy is prevalent in multiple trauma patients and worsens bleeding complications, leading to higher morbidity and mortality rates. Hyperglycemia upon admission predicts hemorrhagic shock and mortality in severely injured patients. This study aimed to assess admission glucose levels as an independent prognostic factor for coagulopathy in multiply injured patients.

**Methods:**

This retrospective cohort study observed multiple trauma patients treated at a level I trauma center between January 1, 2005, and December 31, 2020. Coagulopathy was defined as an international normalized ratio (INR) > 1.4 and/or activated thromboplastin time (APTT) > 40 s. Analysis of variance compared clinical and laboratory parameters of patients with and without coagulopathy. Receiver-operating-characteristic (ROC) and multivariate logistic regression analyses identified risk factors associated with coagulopathy.

**Results:**

The study included 913 patients, of whom 188 (20%) had coagulopathy at admission. Coagulopathy patients had higher mortality than those without (26% vs. 5.0%, *p* < 0.001). Mean glucose level in coagulopathy patients was 10.09 mmol/L, significantly higher than 7.97 mmol/L in non-coagulopathy patients (*p* < 0.001). Admission glucose showed an area under the curve (AUC) of 0.64 (95% CI [0.59–0.69], *p* < 0.001) with an optimal cut-off point of 12.35 mmol/L. After adjusting for other factors, patients with high admission glucose had a 1.99-fold risk of developing coagulopathy (95% CI 1.07–3.60). Other laboratory parameters associated with coagulopathy included haemoglobin, bicarbonate (HCO3), and lactate levels.

**Conclusion:**

This study emphasizes the significance of admission blood glucose as an independent predictor of coagulopathy. Monitoring hyperglycemia can aid in identifying high-risk patients.

## Introduction

Multiple trauma is a complex and heterogenous condition which encompasses a wide range of injuries and represents a leading cause of death and morbidity worldwide [1]. Traditionally, multiple trauma is defined as the occurrence of several simultaneous injuries to different body parts, with at least one or a combination of injuries posing a significant risk to the patient’s life [2, 3]. This condition is influenced by several risk factors, including human behavior, economics, politics, and social health [1, 4]. The impact of trauma extends beyond its medical implications and poses a threat to public health. Despite significant advancements in trauma care, there is still an enormous challenge in understanding the pathophysiological mechanisms associated with traumatic injuries during the early phase after injury. Massive hemorrhage is a leading cause of traumatic fatalities and often leads to coagulopathy, which is marked by the activation of the coagulation and fibrinolytic systems [5]. This condition, known as acute traumatic coagulopathy, can increase the likelihood of multiple-organ failure and mortality. A major challenge associated with traumatic-induced coagulopathy is the limited understanding of its underlying mechanisms, which hinders the development of effective prevention strategies. Severe blood loss can cause hemorrhagic shock and can lead to significant morbidity and mortality. The number of deaths from hemorrhage following physical trauma is estimated to be as high as 1.5 million per year worldwide [6]. In the search for further causes of coagulopathy, serum glucose levels are moving into focus. In order to tackle this predicament, various models have been formulated to anticipate outcomes subsequent to major trauma. For instance, the coincidence of elevated glucose levels with alteration of bleeding tendency in polytrauma mice has already been identified [7, 8]. Furthermore, a retrospective analysis conducted by Kreutziger in 2009 revealed that patients with higher blood glucose levels upon admission had a greater likelihood of mortality from hemorrhagic shock. Subsequently, Kreutziger conducted a further study in 2015, which demonstrated that blood glucose levels at the time of admission could predict the development of hemorrhagic shock in polytrauma patients. Moreover, a study by Winkelmann in 2019 found a positive correlation between blood glucose levels at the point of admission and an increased risk of shock and mortality in polytrauma patients. Despite these findings, the pathophysiological mechanisms underlying coagulopathy and shock after trauma are still not fully understood. This highlights the need for further studies to explore the factors associated with these conditions, including hyperglycemia. Understanding the relationship between hyperglycemia and coagulopathy in polytrauma patients is a crucial research area that can help develop more effective strategies for preventing and treating coagulopathy, ultimately leading to a reduction in associated morbidity and mortality. The purpose of this study was to assess whether hyperglycemia is independently associated with an increased risk for coagulopathy in multiply injured patients. Measurement of blood glucose levels on admission is faster and more readily available than measurement of conventional coagulation parameters. Accordingly, the results of this study may contribute to adopted treatment strategies for preventing and managing coagulopathy, as blood glucose measurement may be able to identify the trauma patients at risk for coagulopathy earlier and reliably. This could also be of great advantage in less developed healthcare systems with limited medical resources.

## Materials and methods

### Study design and patient selection

**Fig. 1:**
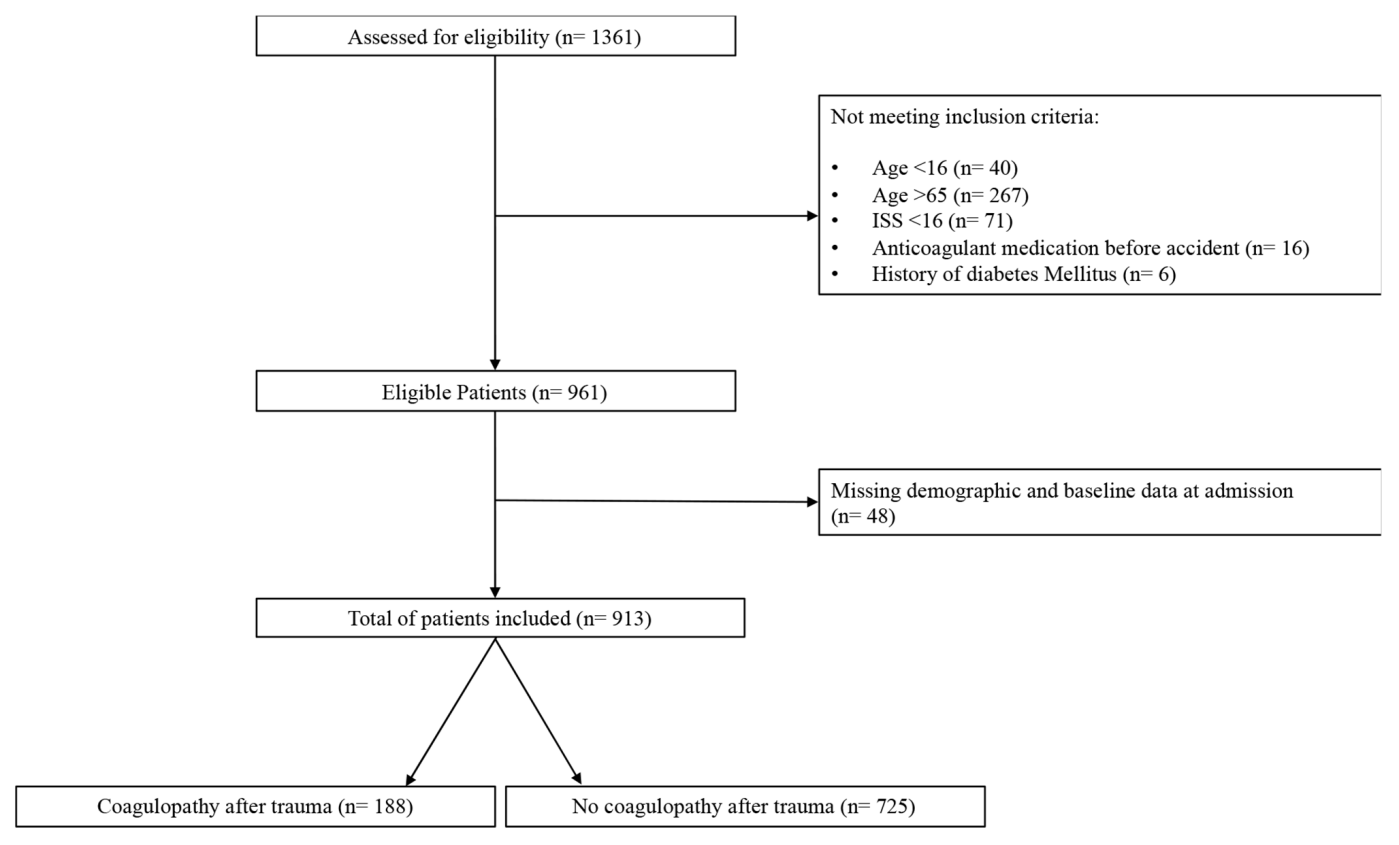
Flow diagram of patient selection

This was an observational, retrospective cohort study which included all primary polytrauma patients (ISS ≥ 16) admitted to the emergency department of a level I trauma center between 1st January 2005 to 31st December 2020 within the first six hours after trauma. Patients aged 16–65 years were eligible for inclusion. Exclusion criteria were: (1) diabetes mellitus, (2) a history of coagulopathy (3) treatment with anticoagulant medication, (4) missing of demographic and baseline data at admission. The process of patient selection is shown in Fig. 1.

### Parameters and definitions

Coagulation parameters including the international normalized ratio (INR) and prothrombin time (PTT; in seconds) were obtained using standard laboratory tests. Additionally, thrombocyte count (x10^9^/L) was also obtained using standard laboratory examination. Coagulopathy was defined as an INR > 1.4 and/or PTT > 40s upon admission.

Shock Index (SI = HR/SBP) was calculated using heart rate (HR; in min-1) and systolic blood pressure (SBP; in mmHg). Laboratory analysis of shock parameters was performed, including measurements of pH value, base excess (BE; in mmol/L), lactate concentration (in mmol/L), bicarbonate level (HCO_3_; in mmol/L), glucose concentration (in mmol/L), and hemoglobin level (Hb; in g/dL). These measurements were conducted using the ABL800 FLEX blood gas analyzer from Radiometer GmbH in Krefeld, Germany. Shock upon admission was defined as SBP ≤ 90mmHg and/or shock index ≥ 0,9.

To control for clinical confounders, data on duration of mechanical ventilation, duration of intensive care and overall in-patient care, as well as transfusion requirements for packed red blood cells (PRBC), fresh frozen plasma (FFP), and thrombocyte concentrate (TC) from patient records were collected. Injury patterns and severity related to specific organs were classified using the 2008 update of the Abbreviated Injury Scale (AIS) [9]. The overall injury severity was calculated using the Injury Severity Score (ISS) [10]. Demographic confounders included age, sex, and mortality.

### Statistical analysis

The statistical analyses were conducted using IBM SPSS software (Version 24, IBM, Armonk, NY, USA). Bivariate analysis was employed to identify predictive variables for hyperglycemia. Differences between patients with and without coagulopathy were assessed using Mann-Whitney tests for continuous variables and the chi-square test for categorical variables. Receiver-operating-characteristic (ROC) analysis and the area under the curve (AUC) were employed to estimate the optimal cutoff values for clinical and laboratory indicators of coagulopathy. The Youden index (J) was utilized to determine the cutoff points that balanced sensitivity and specificity. The diagnostic values considered in this analysis included glucose, lactate, base excess, hemoglobin, pH, and HCO3. To examine the independent effect of glucose on coagulopathy, binary logistic regression analysis was performed. The variables were dichotomized using the optimal cutoff values determined by the Youden index (J). The variables included in the analysis were glucose, base excess, pH, hemoglobin, HCO3, and lactate. The logistic regression models were controlled for age, gender, and Injury Severity Score (ISS).

## Results

### Demographics and coagulopathy

In total, 913 multiple trauma patients were included in the study fulfilling inclusion and eligibility criteria. Among the entire patient sample, 188 individuals were diagnosed with coagulopathy, while the remaining 725 patients did not display any clinical indications of coagulopathy.

The average age of the patients in the study was 37.93 ± 14.09 years. Among the participants, 663 (72.6%) were male and 250 (27.4%) were female. The mean Injury Severity Score (ISS) was 28.80 ± 10.42. At admission, 717 patients (78.5%) presented with shock, and within the course of hospital treatment, 82 patients (8.98%) succumbed to their injuries. Additional clinical and outcome data, as well as laboratory parameters, are summarized in Table (1). Of the entire patient cohort, 188 individuals (20.6%) were diagnosed with coagulopathy upon admission. Bivariate analysis was conducted to compare the characteristics between patients with and without coagulopathy.

Bivariate analysis was conducted to assess differences between patients with and without coagulopathy upon admission. Patients with coagulopathy were significantly older, with a mean age of 39.01 years, compared to patients without coagulopathy, who had a mean age of 33.73 years. Additionally, patients with coagulopathy had a substantially higher Injury Severity Score (ISS), with a mean ISS of 35.15, whereas patients without coagulopathy had a mean ISS of 27.16.

Furthermore, patients with coagulopathy experienced extended periods of mechanical ventilation, averaging 340.28 h, in contrast to the 202 h observed in patients without coagulopathy. The transfusion requirements were notably higher for coagulopathic patients during the initial 48 h following admission, with a mean of 14.68 units of packed red blood cells (PRBC) and 11.92 units of fresh frozen plasma (FFP), in comparison to patients without coagulopathy, who required 3.94 units of PRBC and 2.81 units of FFP.

The mortality rate among patients with coagulopathy was markedly elevated, standing at 26.06%, whereas the mortality rate for patients without coagulopathy was significantly lower at 4.55% (*p* < 0.001).

In addition to these findings, patients with coagulopathy displayed lower levels of hemoglobin, with a mean of 9.33 g/dL, in contrast to 12.58 g/dL in patients without coagulopathy. They also had higher levels of lactate with means of 4.47 mmol/L, compared to 2.59 mmol/L in their counterparts without coagulopathy. Furthermore, coagulopathic patients had a lower body temperature upon admission (34.98 °C) compared to patients without coagulopathy (35.74 °C). These differences were found to be statistically significant, with *p*-values < 0.001. Mean glucose levels were higher in patients diagnosed with coagulopathy compared to patients without coagulopathy (*p* < 0.001) (see Table 1).

**Table 1:** Baseline, demographic, outcome and laboratory data of patients with and without coagulopathy

	Total (*n* = 913)	Coagulopathy + (*n* = 188)	Coagulopathy - (*n* = 725)	*p*
Age [years], mean ± SD	37.93 ± 14.09	39.01 ± 14.01	33.73 ± 13.59	< .001^a^
Male sex, n (%)	663 (72.62)	134 (71.28)	529 (72.96)	0.645^b^
Injure Severity Score (ISS)	28.80 ± 10.42	35.15 ± 13.18	27.16 ± 8.87	< .001^a^
Abbreviated Injury Scale (AIS)				
AIS_head_, mean ± SD	2.19 ± 1.76	2.30 ± 1.91	2.16 ± 1.74	0.372^a^
AIS_face_, mean ± SD	0.84 ± 1.16	0.88 ± 1.17	0.83 ± 1.15	0.584^a^
AIS_chest_, mean ± SD	2.68 ± 1.55	3.21 ± 1.42	2.55 ± 1.56	< .001^a^
AIS_abdomen_, mean ± SD	1.22 ± 1.49	1.70 ± 1.59	1.10 ± 1.43	< .001^a^
AIS_extremities_, mean ± SD	2.20 ± 1.35	2.48 ± 1.44	2.12 ± 1.32	< .001^a^
AIS_external_, mean ± SD	0.72 ± 0.91	0.97 ± 1.07	0.65 ± 0.86	< .001^a^
Mech. ventilation [hours], mean ± SD	230.47 ± 298.87	340.28 ± 36.36	202 ± 275.05	< .001^a^
Intensive care [days], mean ± SD	13.42 ± 13.55	17.39 ± 15.76	12.42 ± 12.74	< .001^a^
In-patient care [days], mean ± SD	23.82 ± 18.94	27.31 ± 23.12	22.91 ± 17.61	0.046^a^
*Transfusion requirements*				
PRBC 48 h [units], mean ± SD	6.15 ± 10.41	14.68 ± 15.52	3.94 ± 7.11	< .001^a^
FFP 48 h [units], mean ± SD	4.68 ± 8.29	11.92 ± 11.50	2.81 ± 5.94	< .001^a^
TC 48 h [units], mean ± SD	0.80 ± 2.06	2.36 ± 3.34	0.39 ± 1.29	< .001^a^
PRBC total [units], mean ± SD	10.89 ± 17.26	22.43 ± 23.83	7.90 ± 13.60	< .001^a^
FFP total [units], mean ± SD	6.60 ± 12.21	15.93 ± 15.79	4.18 ± 9.74	< .001^a^
TC total [units], mean ± SD	1.13 ± 3.35	2.94 ± 4.72	0.66 ± 2.70	< .001^a^
Mortality, n (%)	82 (8.98%)	49 (26.06%)	33 (4.55%)	< .001^b^
Glucose [mmol/L], mean ± SD	8.41 ± 3.37	10.09 ± 4.59	7.97 ± 2.82	< .001^a^
Temperature [ºC]	35.58 ± 1.27	34.98 ± 1.49	35.74 ± 1.16	< .001^a^
Base excess [mmol/L], mean ± SD	-2.52 ± 4.81	-5.30 ± 6.59	-2.02 ± 3.65	< .001^a^
pH, mean ± SD	7.36 ± 1.38	7.26 ± 0.17	7.33 ± 0.09	< .001^a^
Hemoglobin [g/dL], mean ± SD	12.05 ± 3.76	9.33 ± 2.57	12.58 ± 1.92	< .001^a^
Lactate [mmol/L], mean ± SD	2.98 ± 2.45	4.47 ± 3.62	2.59 ± 1.85	< .001^a^
HCO3 [mmol/L], mean ± SD	22.65 ± 4.33	20.54 ± 6.21	23.20 ± 3.48	< .001^a^
Blood pressure [mmHg], mean ± SD	118.29 ± 28.56	106.07 ± 29.05	121.46 ± 27.58	< .001^a^
Heart frequency [min 1], mean ± SD	91.89 ± 21.18	99.85 ± 234.88	89.82 ± 19.61	< .001^a^
Thrombocytes [10^9^/L], mean ± SD	599.56 ± 104.05	214.85 ± 573.87	699.33 ± 110.92	< .001^a^
INR, mean ± SD	1.29 ± 0.63	1.89 ± 1.19	1.13 ± 0.12	< .001^a^
PTT [s], mean ± SD	33.76 ± 22.36	58.83 ± 39.36	27.26 ± 4.94	< .001^a^
Shock, n (%)	717 (78.53)	175 (93.09)	542 (74.76)	< .001^b^

^a^ Mann-Whitney U-test

^b^ Chi-square-test

SD = standard deviation, ISS: Injury severity score, AIS: Abbreviated injury scale, PRBC = packed red blood cells, FFP = fresh frozen plasma, TC = thrombocyte concentrate, 48 h = within 48 h after admission, INR = international normalized ratio, PTT = partial thromboplastin time

### Associations between clinical and laboratory parameters with coagulopathy

**Fig. 2:**
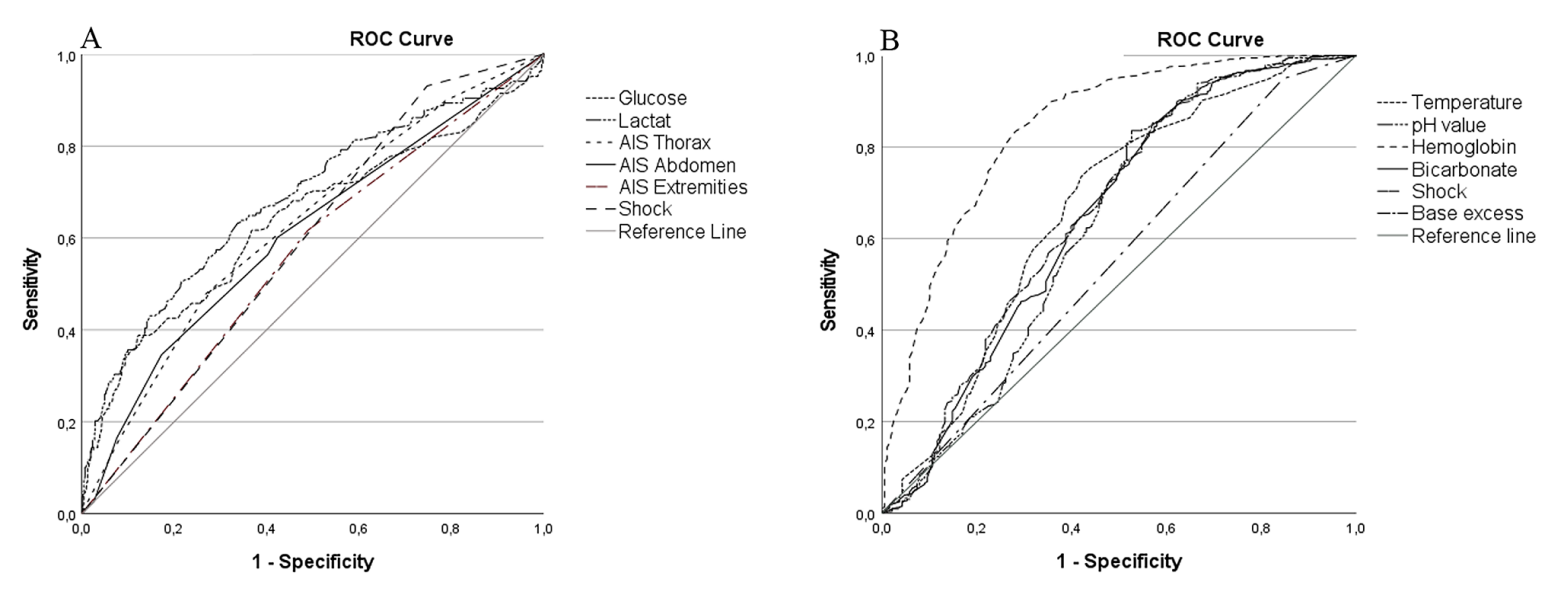
Receiver operating characteristic (ROC) curve and corresponding area under the curve (AUC) with a 95% confidence interval (95% CI) for evaluating the clinical and laboratory indicators of coagulopathy upon admission in patients with multiple injuries **A** Positive correlation parameters; **B** Negative correlation parameters

ROC analysis was conducted to assess the performance of various variables, including glucose, base excess, pH value, hemoglobin, lactate, bicarbonate (HCO_3_), AIS_chest_, AIS_abdomen_, AIS_extremities_, temperature, and shock, in predicting coagulopathy. Positive correlation parameters are presented in Fig. 2A, negative correlation parameters in Fig. 2B. The ROC curve analysis revealed that glucose exhibited an AUC of 0.64 (95% CI [0.59–0.69], *p* < 0.001), with a calculated optimal cut-off value of 12.35 mmol/L and a Youden J of 0.20 for predicting coagulopathy. The sensitivity and specificity of glucose were 27.1% and 92.7%, respectively. The positive predictive value and negative predictive value were 49.0% and 83.1%, respectively. These findings indicate that glucose had the highest specificity but the lowest sensitivity among all laboratory shock parameters. Further details on the ROC curve and cross-tab analyses of laboratory shock parameters are presented in Fig. 2.

**Table:** 

	AUC (95%-CI)	*p*	Cut off	Youden J	Sensitivity	Specificity	PPV	NPV
Glucose	0.64 (0.59–0.69)	< 0.001	12.35	0.20	27.1	92.7	49.0	83.1
Base excess	0.66 (0.61–0.71)	< 0.001	-5.15	0.28	43.1	84.1	41.3	85.1
pH value	0.62 (0.57–0.67)	< 0.001	7.27	0.30	43.6	83.6	40.8	85.1
Hemoglobin	0.84 (0.80–0.87)	< 0.001	10.65	0.55	71.8	83.4	52.9	91.9
Lactate	0.68 (0.64–0.73)	< 0.001	2.79	0.30	63.3	66.2	32.7	87.4
Bicarbonate/ HCO_3_	0.65 (0.60–0.70)	< 0.001	20.10	0.27	42.0	85.4	57.3	58.0
AIS_chest_	0.63 (0.58–0.67)	< 0.001	3.50	0.21	73.1	47.9	84.4	31.6
AIS_abdomen_	0.61 (0.56–0.66)	< 0.001	2.50	0.17	82.8	34.6	83.0	34.2
AIS_extremities_	0.58 (0.53–0.63)	0.001	2.50	0.13	51.0	61.7	83.7	24.6
Temperature	0.66 (0.61–0.71)	< 0.001	35.15	0.32	26.6	42.0	63.8	12.9
Shock	0.41 (0.37–0.45)	< 0.001	NA	NA	25.2	93.1	93.4	24.4

Determination of optimal cut-off value using Youden Index (J) with sensitivity, specificity, positive predictive value (PPV), and negative predictive value (NPV)

To analyze whether the clinical and laboratory parameters had an independent effect on the probability of coagulopathy, multivariate binary logistic regression was performed. Odds ratios and 95% confidence intervals were calculated. Our findings indicate that patients with glucose levels exceeding 12.35 mmol/L have a significantly higher risk of developing coagulopathy (OR = 1.99, 95% CI 1.07–3.60). This suggests that such patients have a 99% greater chance of experiencing coagulopathy. Regarding other laboratory parameters, lower values of hemoglobin (OR = 0.09, 95% CI 0.05–0.13) and HCO3 (OR = 0.47, 95% CI 0.22–0,83) (according to cut off values) significantly predict coagulopathy. Furthermore, lactate (OR = 2.01, 95% CI 1.31–3.12), was also identified as a positive predictor of coagulopathy. The degree of severity of the injuries (ISS) proved to be a positive predictor of coagulopathy (OR = 1.04, 95% CI 1.02–1.06). In addition, the presence of shock was also a positive predictor for the occurrence of coagulopathy (OR = 2.39, 95% CI 1.17–4.86) as shown in Table (2).

**Table 2:** Results of multivariate logistic regression analysis with odds ratio (OR) and 95% confidence interval (95% CI) of clinical and laboratory predictors of coagulopathy in multiply injured patients

	OR (95% CI)	*p*
Age (per year)	0.96 (0.95–0.98)	< 0.001
Sex (male/female)	2.08 (1.26–3.44)	0.001
ISS (per point)	1.04 (1.01–1.06)	0.009
Glucose (≤ 12.35 to ≥ 12.35 mmol/L)	1.99 (1.07–3.60)	0.039
Base excess (≤ -5.15 to ≥-5.15 mmol/L)	0.93 (0.45–1.91)	0.445
pH (≤ 7.27 to ≥ 7.27)	0.71 (0.40–1.25)	0.221
Hemoglobin (≤ 10.65 to ≥ 10.65 g/dL)	0.09 (0.05–0.13)	< 0.001
HCO3 (≤ 20.10to ≥ 20.10 mmol/L)	0.47 (0.22–0.83)	0.016
Lactate (≤ 2.79 to ≥ 2.79 mmol/L)	2.01 (1.30–3.13)	0.002
AISchest	1.15 (0.70–1.93)	0.582
AISabdomen	1.18 (0.74–1.99)	0.537
AISextremities	0.77 (0.49–1.19)	0.255
Temperature Shock	0.44 (0.27–0.64) 2.39 (1.17–4.86)	0.417 0.017

## Discussion

Coagulopathy is a serious medical condition that affects the ability of blood to clot and is prevalent within trauma patients. Early detection and management of coagulopathy are crucial for improving the prognosis of multiply injured patients, as coagulopathy is associated with poor prognostic outcomes, such as shock and mortality [11].

The primary aim of this study was to investigate the relationship between hyperglycemia and coagulopathy in patients with multiple injuries. The findings of the study revealed that patients with admission glucose levels ≥ 12.35 mmol/L had a 1.99-fold higher risk for developing coagulopathy. Moreover, other laboratory parameters, including hemoglobin, bicarbonate, and lactate levels, were identified as positive predictors of coagulopathy. Glucose demonstrated a superior prognostic performance compared to hemoglobin, bicarbonate, pH, and base excess (with an area under the curve of 0.64 (0.59–0.69) and odds ratio of 1.99 (1.07–3.60). Only lactate exhibited similar or slightly better prognostic value for the presence of coagulopathy. The degree of injury severity and the presence of shock were also found to be positive predictors of coagulopathy. Furthermore, patients with coagulopathy experienced more severe outcomes, including higher mortality rates, prolonged stays in the intensive care unit, and higher transfusion requirements. The elevated mortality rates observed in the coagulopathy group (26%) can be attributed to several significant factors. Patients in this group had a notably higher mean Injury Severity Score (ISS) of 35.15, indicating more severe overall trauma. Additionally, they exhibited higher Abbreviated Injury Scale (AIS) scores in critical injury regions, such as the head, chest, abdomen, and extremities, highlighting the extent and severity of injuries. Furthermore, patients with coagulopathy required substantial transfusions within the initial 48 h post-admission, including packed red blood cells (PRBC) and coagulation factors like fresh frozen plasma (FFP) and thrombocyte concentrates (TC). This heightened need for transfusions, often termed massive transfusion, signaled significant bleeding or critical hemorrhage [12–14]. The coagulopathy group also had a slightly older average age of 39.01 compared to 33.59 for patients without coagulopathy, which may contribute to increased mortality due to potentially reduced physiological reserves in older individuals following trauma. Lastly, these patients experienced prolonged mechanical ventilation during their intensive care unit stay, averaging 340.28 h compared to 202.00 h for patients without coagulopathy. This extended ventilation duration may indicate more severe respiratory complications and organ dysfunction. In summary, the heightened mortality in the coagulopathy group stems from a combination of factors, including the severity of injuries, increased transfusion requirements, slightly older age, and prolonged mechanical ventilation.

These findings are consistent with previous research that supports the association between admission glucose and incidence of hemorrhagic shock as well as mortality in multiply injured patients [15, 16]. The results of this study align with the findings of a previous investigation, which identified hyperglycemia as an autonomous risk factor for coagulopathy in cases of isolated open traumatic brain injury [17]. Further studies have already investigated glucose has an independent predictor for increased intensive unit and overall hospital stay [18]. Similar studies also investigated the changes of glucose levels upon the first hours of admission to help predict hospital mortality [19]. However, to the authors’ knowledge, this is the first study analyzing admission glucose levels as an independent predictor for coagulopathy in multiple trauma patients.

While the study provides valuable insights into the relationship between glucose levels and the development of coagulopathy, there are limitations that need to be considered when interpreting the results. Firstly, the presented study is a retrospective analysis with its inherent limitations. Additionally, a significant number of patients (33%) had to be excluded due to missing data. Although this is comparable to previously published studies [11, 15–17], missing data can be misleading and might influence risk prediction [20]. The cross-sectional design of the study also restricts the gathering of data to a singular moment in time, making it impractical to determine any longitudinal patterns or alterations over an extended period. Furthermore, this was a single-center study. All patients in this analysis were treated in a level I trauma center in a high-income country with a sophisticated trauma system. Our findings might not be able to be generalized to other contexts. Further studies are required to validate our results in diverse settings. Another potential limitation of our study is that our definition of coagulopathy focused primarily on abnormalities in the plasma coagulation system, as indicated by an INR > 1.4 and/or PTT > 40 s, while thrombocytopenia was not included in our definition. Finally, the definition of coagulopathy lacks a unified standard, and future studies may require a unified definition.

In our study, we favored the traditional Injury Severity Score (ISS) over the newer Berliner Definition of Polytrauma [3, 10, 21, 22]. This choice was based on ensuring comparability with prior research and to sidestep potential pitfalls such as circular reasoning and selection bias, given the Berliner Definition’s inclusion of coagulopathy—a key focus of our study. However, we acknowledge the Berliner Definition’s comprehensive approach, capturing a multifaceted understanding of trauma. One primary consideration was to circumvent the potential for circular reasoning within our research design. The Berliner Definition of Polytrauma includes coagulopathy as one of its defining parameters. Given that our primary research focus was to investigate the impact of glucose on coagulopathy, adopting the Berliner Definition would have introduced a methodological challenge. This would have resulted in a circular logic scenario, where we would essentially be studying the influence of a variable that is partially characterized by the presence of our primary outcome. Moreover, we aimed to minimize the risk of selection bias by not including coagulopathy as a pre-defined component of our trauma severity criteria, as is the case in the Berliner Definition. By incorporating coagulopathy into the trauma severity criteria, the Berliner Definition could inadvertently lead to the selection of patients already predisposed to coagulopathy. Analyzing the relationship between glucose and coagulopathy within this preselected subgroup might artificially magnify any observed associations due to this inherent bias. While the ISS was more fitting for this study, future research might find the Berliner Definition’s nuanced approach advantageous.

Despite these limitations, the study’s findings could still inform clinical decision-making and lead to improved patient outcomes. As an independent predictor of coagulopathy, monitoring blood glucose level could be an opportunity to detect and manage coagulopathy early, thus improving the prognosis of multiply injured patients. The advantage of glucose measurement lies in its wide availability, even in less developed regions, and its suitability for point-of-care diagnostics [23]. It is essential to continue the research on coagulopathy for better understanding of its causes and risk factors, allowing improved detection, management, and clinical decision-making for optimal patient outcomes.

## Conclusion

The present study underscores the correlation between elevated glucose levels and an elevated risk for coagulopathy development in severely injured patients. The significance of measuring admission blood glucose as a readily accessible bedside test is highlighted by these findings. When combined with other parameters, this test has the potential to assist in identifying multiply injured patients who are at a heightened risk of developing coagulopathy. Moreover, the advantage of glucose measurement lies in its widespread availability, even in less industrially developed countries, and its utility as a point-of-care diagnostic tool.
